# The Fate of Associations

**Published:** 1897-01

**Authors:** J. C. Walton

**Affiliations:** Howell


### PROCEEDINGS.

### The Fate of Associations.

BY DR. J. C. WALTON, OF HOWELL.

Read at the Michigan Association.

Associations of individuals for mutual help and encouragement existed in ancient Egypt, flourished in Greece and Rome, and exerted an influence in all European countries during the Middle Ages. There were Danish, German and Belgian guild laws which provided that no one should become a member who had "dirty hands" or "blue nails" or "who hawked his wares on the street." Concord and ethics were assured by stringent regulations, and no member was allowed to entice away a guild brother's customers. But principles of exclusiveness were introduced and exerted tyrannic sway. Privileged cliques grew oppressive. Petty rivalries and hateful egotisms took the place of the great idea of association until they lost their original characteristics and became merely social and benevolent clubs.

Topics of a medical nature were first discussed in the Royal Society of England in the 17th century, and medical societies, as we know them, were first formed in the early part of the present century.

Ambitious dentists formed the Society of Dental Surgeons in 1841, the object being to stimulate and encourage investigation, contribute knowledge gained by study or experience to the society, to increase the knowledge and improve the practice of each, and benefit humanity at large. At this time dentistry was in a chaotic condition, with no literature, colleges or societies. Those considered eligible numbered about one hundred in the United States and Canada, and at the first meeting only thirteen were present. For several years the proceedings were instructive, harmonious and dignified, indicative of close study and careful observation, and conducive to a spirit of energy and liberality. Then quibblers and politicians ruled its management and it passed out of existence in 1856.

The American Dental Convention was organized in 1855. Communistic ideas prevailed and an attempt was made to "level up" the profession by admitting the rank and file to an equal share in association benefits. But many exclusive, though able, spirits refusing to participate or be present on equal footing with mediocrity, the association waned in vigor from year to year and passed out of existence in 1880.

The American Dental Association was organized in 1859. The delegate system adopted appears to have been a happy plan for encouraging general association work, for State and local societies have rapidly multiplied until, to-day, we have over one hundred. It has never, however, been accepted fully as the national organization its founders aimed at, and of late, evidences of enfeeblement are apparent, and confidence in its future influence begins to decline.

The original State Society of Michigan was started in 1855, and reorganized under a charter October 11, 1876.

Article II of our constitution reads: "The object of this Association shall be to cultivate the science and art of dentistry and its collateral branches, to elevate and sustain the professional character of dentists, to promote among them mutual improvement, social intercourse and good feeling, and collectively, to represent and have cognizance of the common interests of the dental profession in the State."

The Michigan Dental Association has served a good purpose and filled its office in influencing the thoughts and actions of its members. Its management has been in the hands of those of high purpose, unquestioned zeal, discriminating judgment—men accustomed to exercise important generalship. Notwithstanding this fact, however, and also that so many of our brightest dentists have drawn their happiest inspirations from its meetings, our roll does not show a complimentary membership. With over eleven hundred dentists in Michigan, our State Society has a membership of only one hundred and forty. Why is this? It has, in the usual form, invited to membership those considerd eligible by the prevailing sentiment of society attendants. Few, I fear, appreciate the hard work done and self-sacrifice practiced by our officials to make this meeting the success it is, and yet, only about one in ten of our dentists are present. Why not analyze the situation and try to comprehend its meaning?

The apathy of societies in general is being remarked. The failure to reach the profession, as a whole, and through them, win of the world a better appreciation of our art, is being mourned. We believe that successful results for general welfare can be secured only by general effort, but melancholy has been the failure to unify and associate for the general good. Twenty thousand dentists in the United States have no interest in associations. Perhaps five thousand have. Those only who aspire to social rank in the profession bother themselves about societies.

The laws of human impulse are the same to-day is in guild-craft days.

We are drifting from some of the acceptable objects of organization. We may be "cultivating the science and art of dentistry," but we are not trying very hard "to promote mutual improvement, social intercourse and good feeling" among dentists in general. Society-goers, as a rule, are trying "to elevate and sustain the professional character" of society members, but nearly nine-tenths show their appreciation of our efforts to "represent and have cognizance of the common interests of the profession" by non-support and non-attendance. Are not principles of exclusiveness gaining ground? Are not cliques monopolizing the honors? Are not petty rivalries and hateful egotisms taking the place of the original ideas of association? Does not the history of associated efforts show that when "the greatest good to the greatest number" is the ruling sentiment associations flourish, and that they wane when the masses are forgotten?

We are constantly blaming those outside our societies for the attitude in which we find them, when there are reasons that show they are less blameworthy than we. Half our dentists do not understand the object and advantages of association. A

strange confusion prevails in regard to the duties of the State Board of Dental Examiners, the aim of the State Society, the provisions of the dental law and the responsibility for its enforcement. With nearly fifty colleges, twenty journals and a hundred societies it is hard to explain the misunderstanding and misinformation existing.

Cities dominate in society control, but do not breed liberal and broad-minded men who are capable of seeing the profession as a whole. Cities produce specialists and specialists favor what will tend to promote the success of their specialty. The mass of suburban and rural dentists do not, and can not practice as specialists. Accepted association etiquette is too often a brand of professional piety that blushes at communion with rustic manners and shop-talk. Mediocrity is rarely forgiven and provided for. The country dentist expects, and is expected, to submit to the specialty interests and club fashionableness of urban management. The average dentist is in a position to feel that fellowship can not be accorded him until he changes, and if he changes, his untutored patients will have little use for him. Therefore, he stays away, living on in solitude, with dwindling interest in professional advancement, too often nursing the belief that professional life, if typified by the vanguard, is but a school of hypocrisy, where a few expend their genius in counterfeiting medicine and many try to conceal, under borrowed dignity, the tooth-carpenter, tooth-jeweler and the maker of store-teeth. The temper of the profession at large is little understood and not studied. We are wofully divided by ethical and other antipathies, and strained relations exist between the society-goer and the non-attendant. Differing dentists are constrained to adopt different means of attracting public attention, suited to the mental, social and financial condition of the clientele desired. Most of us follow it as a vocation with general net results as the incentive. Half, perhaps, are influenced, to a greater or less degree, by the code of ethics and half are indifferent, while a small percent hoist the black flag of dental piracy.

There should be a method higher in popular efficiency than that which qualifies each invitation to its membership, by adding

"if you are a reputable dentist"—that is forever seeking to establish a line of demarkation between its own perfection and the ethical infidelity of "the other fellow." There is wisdom in insisting by one's manner that every one who takes your chair is chaste, moral, honest and intelligent enough to appreciate your effort. Just so in society management. It will prove profitable to give every practitioner credit for being moral, honest and intelligent enough to belong to the Michigan Dental Association until proved unworthy. If the candidate is legally qualified to practice and promises to be governed by the rules of the society, it is enough. No one should be subjected to the insinuating inquisitorial style of examinations that has prevailed in past years.

The individual who desires friends must prove himself a friend. It is so with societies. They must be managed to compensate those whom we wish to attract. To gather in and supply incentive, instruction, and entertainment to the dentists of the State, is the practical problem for State societies. It is not the place to encourage a few to believe in their professional virtue. It should supplement the college, the journal, and stimulate home study. It should be the special agency for the supervision, extension and development of the profession as a whole. To secure excellent legislation and prevent vicious legislation; to help remove apprehensions and correct false ideas; to gather into reports the nebulous information existing among the practical men of our time; to assert the features of an educational force controlled by wise men, is a possible result.

All want to know how to diminish failures, to promote co-operation, to facilitate transmission of ideas to our clientele, to secure proper status, to guard against financial loss—such would be general progress.

We may have the benefit of contest between man and man, which is always a powerful attraction, but it should only be a contest in research, experiment and teaching. It is right to compete with and combat quackery, but wrong to ignore and shun the quack. The way to obliterate darkness is to let in the light. The way to correct newspaper deception is to supply newspaper education. Quackery should be regarded as misdirected energy, and a thoughtful effort made to get control of it. If we are not willing to tolerate it, to listen to it, to find out the truth that may be in it, to supplement it, to enlighten it, we belong back in the witch-burning period of history. We may be sure there is some reason for its existence. Why ignore a reason, wherever found? The savage remains the savage—the thoughts of the savage continually being within him. Place him in the midst of civilization; give him new thoughts, new hopes, new aspirations, new impulses, and you raise him above the savage state. This is the whole history of civilization, of enlightenment. Three out of four of us thus need to be placed in the midst of professional civilization; we need to be given new thoughts, new hopes, new aspirations, new impulses, and raised above ourselves. Ignorance and enmity are convertible terms. Rancor and retaliation are likely to make trouble until transformed by concession and education. Increased knowledge has never brought aught but blessings to the world. Membership in this Society would strengthen any dentist's attachment to his profession. In every State is needed one or more societies that are founded on those broad principles which find their counterpart in the heart of every dentist; a society instituted and managed for no personal or selfish gain; a great school of comparative dentistry, to bring different methods and men in contact and conference; to encourage and deepen the spirit of brotherhood; to emphasize the distinctive achievements of the art; to bridge the chasm of ethical separation between the liberal and the exclusive elements and induce dentists to pull together for our common improvement.

There is little use in cultivating sentiment against the dentist of the masses, refusing to educate the masses and then expecting him to fraternize on present society terms. It is the business of societies to teach and demonstrate the beauties of an ethical life, the profit of correct practice, and the nobility of our calling. If a college education and society affiliations do not develop courtesy for the opinions of others, toleration, a spirit of helpfulness, and an earnest desire to do one's best, they fail of a high mission.

Electricity sleeps in mere potentiality, as a force hid in the coal bin, until, through heat and motion, under proper control, it appears to work its wonders in your office, so the genius of human power lies dormant until shocked into efficiency by a wave of suggestion or a flash of illumination. At this meeting a single word, the turn of a phrase, a demonstration, may be potent to stir into activity a native, dormant power. Who would deny any member of our profession the inspiration? Every dentist should experience the thrill of such an inspiration. Who of you have attended a meeting without having had the deepest wells of your thought strangely moved to action, and going home with energy awakened in every fiber have not felt irresistibly the impulse to do better? Yes, the present age of enlightenment demands this reciprocal action—this interchange of intellectual currents between dentist and dentist. Wisdom so inspired has given to the world all that is worthy in art, science, letters and all else that is imperishable in civilization.

Perhaps most of us think we comprehend the object of association, and the results are satisfactory. If we are right, then no change would be salutary. If the theory is correct, that to level men we must level downward, and that a society should be a professional despotism in which the wisest and ablest are supported by an admiring and sympathetic following, then it is trespassing on your kind forbearance to present this theme.

But, for me, the spirit of this society's action should be altruistic. The terms of admission should be really inviting, not nominally so. The welcome should be generous, not repelling. The rank and file would thus be encouraged and stimulated to self-improvement. Clubs and coteries would still adopt methods to protect their exclusiveness. With them, social qualifications may rightly continue to influence the choice of membership. In a popular body this can not be, and a State Society should be a popular body.

The discussion of the paper (by Dr. Walton) was opened by Dr. Metcalf, as follows:

Mr. President and Gentlemen: I do not suppose, for our present purposes, that it will be necessary to discuss this very excellent paper in all the propositions that it contains; I think, perhaps, as we are a little crowded for time, we had better only, at present at least, look at such parts of the paper as may be interesting and applicable for the present to the effect on our own Society, and I do not suppose, Mr. President, that you will expect me to antagonize this paper unnecessarily; I should dislike to do that, because, with one or two exceptions, I agree with the propositions that have been made in the paper, and so I think it will be sufficient, perhaps, if we discuss the points made which may, in any way, affect this Society.

As stated in the paper, this Society was formed in 1855, and so far as I know, I am the only person here who was present at the formation. The first meeting of this Society was held in the city of Detroit, in the office of Drs. Whiting and Benedict, some time during the year 1855, I think in the autumn of that year. Of course this may seem to you like a very bold statement, coming from one so young and fresh as myself, but I believe it is a fact; and I think I am the only one who was present at that time who attends the meetings of this Association. In fact, there were only about a dozen of them. My impression is that Dr. Whiting, beside myself, is the only one of that little number who has not long since gone down into the shadow.

The organization of that Society was, as stated in the paper, for mutual improvement, and the object as stated in the first call (the call was issued by Drs. Whiting and Benedict) was for mutual improvement first, and next, that the dentists of the State might become acquainted with each other; and thirdly, that we might co-operate in some way to establish a fee-bill that should be general throughout the State. As I said before, the first meeting was attended by, I think, less than a dozen persons, but that was the nucleus for this Society, which, I believe, has been a very successful one, and, so far as I know, as good and as valuable a Society for the dentists of the State as any State's Society in the United States.

Now then, about the fate of this Society. The older dentists present know what the condition of dentistry was at that time, and those who came into the profession years after, by comparing the papers that were read at that time and the papers that have been read here to-day, can see what this Society has accomplished. Then our thoughts and ideas and our dental education were just about as crude as the instruments we then used. Every dentist was instructed in the manufacture of his excavators and the pluggers—they were very crude, and I have, to-day, some of the pluggers that I made many years ago out of excavators, and with leather handles. From that time until this the improvement, not only in the personnel, but the improvements in the papers, the improvement in the operating, the improvements in every detail of dentistry have been very great, and this has all been brought about by Associations in the different States, and I don't know of any Association that has done more for the elevation of the profession than this one; and, although not an operating dentist to-day, I am proud of the personnel of the dentists here in this State, and when I speak of the dentists of this State, I speak of those who attend the meetings of this Association.

I infer from the conclusion of the paper, that some effort ought to be made to open wider our doors and take in more of the dentists of the State, but I am afraid if this were done the best dentists of this Society would step out. We have in the Society the best dentists of the State, and I do not think we want to gather in "dead wood."

It is not only the improvement in appliances and in the papers that are read and the education of the dentists, but what more have we accomplished in the State? We have a dental college second to no other in the world. And how has this been brought about? It could not have been except for this association. We have a dental law requiring that all dentists who enter the practice in this State shall be graduates of our university or of some other dental institution that is regular, and has a curriculum equal to that of the Dental Department of the University of Michigan. Of couse,· you may say, as I have heard it said, that our dental law does not amount to anything, that dentists come to this State and practice and they are not disturbed, but that is a question that will come up after we hear Dr. Corbin's paper, and I will not discuss it further than to say that if you will show me a law upon our statute books, any law, that is enforced, you will show me something that I have never seen. We have a law in this State—and it was secured by this society —that has given us a Board of Dental Examiners; a good deal of fault, I know, has been found from time to time with the board. Of course, while I was a member of it I could not step into the breach and defend them bcause I would be defending myself, but I stand here ready to-day to defend that board, if it is necessary, against any accusation that can be brought against it from any source. It has, so far as I know—and I have been very well acquainted with the board from the very start until to-day—performed its duties manfully and thoroughly. But, I hear some of you say: "What is the use of a board unless it attends to these prosecutions?" Now, every dentist ought to know that it has nothing whatever to do with prosecutions; its only duty is to examine those who come before it and who want to practice in this State, and if they are qualified to give them a certificate of qualification, and if they are not, to reject them, and they have been very thorough in that business. The condition of things that we are enjoying here to-day could not have been accomplished without associated effort. I read, somewhere, of a great fountain, and standing up in the basin of the fountain was a huge figure representing Wisdom with its hands spread out and the waters pouring from each finger in drops below, and in the sunlight it looked like so many crystals falling from the hands of Wisdom, and so it is with this association, it can be likened to this figure of Wisdom—the hands of this association are spread out and these papers and discussions are the drops of wisdom that come forth and we can gather and profit by them.

